# Economic evaluation of cardiac magnetic resonance with fast-SENC in the diagnosis and management of early heart failure

**DOI:** 10.1186/s13561-019-0229-7

**Published:** 2019-05-23

**Authors:** John E. Schneider, Ivana Stojanovic

**Affiliations:** Avalon Health Economics, 26 Washington Street, 3rd Floor, Morristown, NJ 07960 USA

**Keywords:** Heart failure, Cardiac magnetic resonance imaging, Cost-effectiveness, Hospital value analysis, Markov model

## Abstract

**Introduction:**

Heart failure (HF) is a major public health concern, prevalent in millions of people worldwide. The most widely-used HF diagnostic method, echocardiography, incurs a decreased diagnostic accuracy for heart failure disease progression when patients are asymptomatic compared to those who are symptomatic. The purpose of this study is to conduct a cost-effectiveness analysis of heart failure diagnosis comparing echocardiography to a novel myocardial strain assessment (Fast-SENC), which utilizes cardiac-tagged magnetic resonance imaging.

**Methods:**

We develop two models, one from the perspective of payers and one from the perspective of purchasers (hospitals). The payer model is a cost-effectiveness model composed of a 1-year short-term model and a lifetime horizon model. The hospital/purchaser model is a cost impact model where expected costs are calculated by multiplying cost estimates of each subcomponent by the accompanying probability.

**Results:**

The payer model shows lower healthcare costs for Fast-SENC in comparison to ECHO ($24,647 vs. $39,097) and a lifetime savings of 37% when utilizing Fast-SENC. Similarly, the hospital model revealed that the total cost per HF patient visit is $184 for ECHO and $209 for Fast-SENC, which results in hospital contribution margins of $81 and $115, respectively.

**Conclusions:**

Fast-SENC is associated with higher quality-adjusted life years and lower accumulated expected healthcare costs than echocardiogram patients. Fast-SENC also shows a significant short-term and lifetime cost-savings difference and a higher hospital contribution margin when compared to echocardiography. These results suggest that early discovery of heart failure with methods like Fast-SENC can be cost-effective when followed by the appropriate treatment.

**Electronic supplementary material:**

The online version of this article (10.1186/s13561-019-0229-7) contains supplementary material, which is available to authorized users.

## Introduction

Heart failure (HF) is a major public health concern, with a prevalence of 1–2%, or more than 5.8 million people in the U.S. and over 23 million worldwide [1, 2]. Each year in the U.S., more than 550,000 individuals are diagnosed with HF for the first time [3]. The increasing prevalence is due in part to the aging of the population, increasing rates of obesity and diabetes, and more generally the prolongation of the lives of cardiac patients [4–7]. The lifetime risk of developing HF is about 20%, and that risk is the same at age 40 and age 80 [8].

There are approximately one million annual hospitalizations in the U.S. due to HF [4]. Heart failure is the leading cause of all hospitalizations and readmissions in older people, and affects 6–10% of people over the age of 65 [9]. After hospitalization, HF prognosis generally worsens [10]. Although the outcomes for ambulatory HF patients with a reduced ejection fraction have improved with the innovation of drug and device therapies, hospitalized HF patients continue to experience high post-discharge mortality and readmission rates [4]. Heart failure is also associated with a disproportionately higher use of ambulatory services, including emergency department and clinic visits, especially towards the end of life [8, 11, 12]. Nonetheless, many of HF-related hospitalizations can be considered “avoidable”, either due to poor disease management generally or lack of “systems approach” to early diagnosis and treatment (e.g., providing adequate outpatient treatment to avoid hospitalizations) [13–17]. As such, the economic burden of Stage B HF is high and will likely continue to grow over time.

Echocardiography, as the most widely-used diagnostic method, measures left ventricular (LV) ejection fraction (EF) as a global index of ventricular systolic function. However, LVEF is affected by the ventricular geometry and loading condition which may remain unchanged in affected patients until the underlying disease process is advanced [18]. Moreover, LVEF, by itself, has been shown to be a poor indicator of HF disease progression, with a positive predictive value of only 72% [19]. The key to diagnosis of Stage B HF is evidence of cardiac structural remodeling or functional abnormalities in the absence of any HF symptoms [20].

There is recent and developing literature describing the clinical utility of myocardial strain assessment using cardiac-tagged magnetic resonance imaging (CMR) in conjunction with algorithms and software to augment the clinical utility of CMR [21]. For example, strain-encoded CMR (Fast-SENC, known commercially as MyoStrain™ software developed by Myocardial Solutions, Inc., Raleigh, NC) is a heart assessment test that provides myocardial strain measurements within 10 min without using contrast or radiation, and quantifies myocardial strain across 37 regions of the heart that show heart dysfunction on both a global and regional level. By quantifying strain metrics and visualizing the affected regions of the heart, Fast-SENC can identify dysfunction resulting from a broad range of cardiac diseases, including stenosed blood vessels, ruptured atherosclerotic plaque, or microvascular diseases, allowing physicians to make more informed clinical decisions [22]. Fast-SENC also offers previously unavailable cardiac function data to determine the subclinical effects on the heart of HF and associated treatments, allowing physicians to make more timely and effective treatment decisions. In this study, we conduct a cost-effectiveness analysis of HF diagnosis using Fast-SENC, assessing the economic impact of the improved diagnostic capability of Fast-SENC, taking into account changes in the clinical pathway and resources associated with “Fast-SENC-guided” HF treatment compared to the standard of care (i.e., echocardiography).

## Methods

We developed models from the perspectives of payers (i.e., insurance plans) and from the perspective of purchasers (i.e., hospitals and health systems). The payer perspective model is a cost-effectiveness analysis that follows methods recommended by the U.K. National Institute for Clinical Excellence (NICE) as well as guidelines developed by the International Society for Pharmacoeconomics and Outcomes Research (ISPOR).Given insurance switching by patients over time, payers covering initial costs may benefit at all any downstream cost offsets [23, 24]. Consequently, we composed an insurance payer model of a 1-year short term model and a lifetime horizon long term model.

Patient population lifetime costs were modeled with Markov processes. The hypothetical patient cohort is on average 64 years and its characteristics are based on a clinical trial cohort with a pretest probability for early HF of 20%, as determined by a Charlson Comorbidity Index score [25, 26]. The hospital model is a 1-year model and the hypothetical patient cohort matches that one of the payer model.

### Payer model

We developed the HF model framework based on information obtained from a review of the published literature containing information on the management and clinical outcomes of HF patients. The model assumed patients will undergo the standard HF screening assessing patient history and physical examination. Scoring three or higher on the Charlson Comorbidity Index scale during the initial examination, would add a diagnostic assessment of the heart with either echocardiography (ECHO) or Fast-SENCT to assess the long-term effects of early HF diagnosis with imaging modalities, the model accounted for a lifetime of costs and health effects across a hypothetical cohort of patients using a Markov model of HF disease progression [27–33]. The model used transition probabilities (shown in Additional file 1)to allow patients to transition between disease states over time. Each disease state was assigned a time-dependent utility and cost. Disease state transition probabilities are based on literature and consultations with clinical experts. Based on discussions with clinical experts, we assumed that the initial diagnostic effect is associated with an initial treatment effect, and that the initial treatment effect impacts subsequent disease state transition probabilities (i.e., through better protection of heart muscle during the episode of care following diagnosis).

The long-term expected health effects and costs of imaging modality strategies were calculated by multiplying the total time spent in each disease state by the utilities and costs corresponding to these states, respectively. The model employed 1-year time cycles and the simulation stopped when all patients in the hypothetical cohort died. The simulation begins with a short-term model that divides HF patients based on the imaging modality. The group that got early HF diagnosis with Fast-SENC or went undiagnosed in the ECHO group. With a diagnostic accuracy of 100%, Fast-SENC allowed for patients to be treated with medications and recommended lifestyle modifications, reducing the incident rate of HF hospitalizations [16]. ECHO is the imaging standard of care in assessing early deterioration of LV function, however it is not able to detect dysfunction within the heart wall. Thus, ECHO will fail to diagnose the early signs of HF.

**Fig. 1:**
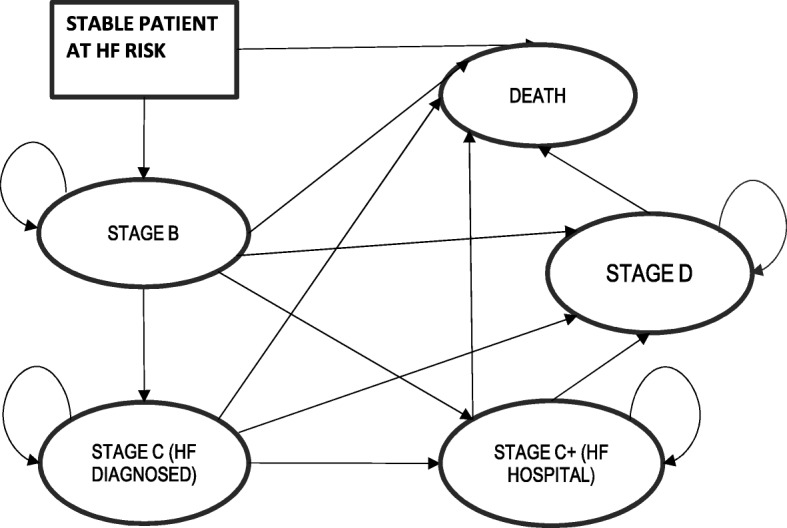
Markov model representing HF disease progression

The Markov decision analytic model was structured with five disease states, including the beginning state of HF (Stage B), Stage C, Stage C+, Stage D and the death state as described in the ACCF/AHA Guideline for the Management of Heart Failure (Fig. 1) [34]. In the model not all patients started in the early HF Stage B state, as some patients were assumed to have already had a HF hospitalization and thus started in the Stage C. Individuals without HF at the time of the screening stayed in the suspected HF stage, (i.e., Stage B) until they developed HF and progressed further into one of the more advanced Markov disease states. Patients could then be detected by the subsequent annual screening similar to the individuals with HF who were missed with the initial screening. Finally, all individuals could die from causes unrelated to HF, while HF patients could also die due to HF. In the model, health effects were determined by calculating expected life-years and quality adjusted life-years (QALY). Utilities for each of the five disease states were based on the HF patient cohort reported by Banz et al. [35]

.

### Data

Fast-SENC is a relatively new technology, therefore clinical data required to populate the model was collected from the HF literature, including health statistics and expert opinions. Data inputs for the payer are shown on Table 1, and data inputs for the hospital model are shown on Table 2. The expected treatment effect for patients who are diagnosed with early HF and treated with beta-blockers and ACE inhibitors was estimated from a clinical study reported by Pfeiffer et al. The risk of death from causes other than HF based was based on age- and gender- life tables for the U.S. individuals, whereas risk from death from HF was estimated based on the study of 3752 individuals in the Cardiovascular Health Study [25]. The costs taken into account in the model included HF hospitalizations, annual pharmacology costs, annual office visits, imaging, and procedural intervention costs, shown in Tables 1 and 2. Utility values were also based on the literature and assigned to each disease state by the New York Heart Association (NYHA) classification based on clinical guidelines [35, 36]

**Table 1:** Clinical Input Data for Payer Model

Variables	Base-case	Article References
Average age of cohort	64	[25]
Prevalence of HF stage B+ after Charlson screening	0.2	Pretest assumption
Outcomes discount rate	3%	[40]
SENC Rest Diagnostic Accuracy	1	[18]
4-year progression in prevalence of stage B	0.154	[41]
ECHO at rest		
Stage C	4.27%	[42]
Stage C+	4.55%	[42]
Stage D	4.50%	[26]
Mortality	6.94%	[42]
Fast-SENC		
Stage C	3.11%	[42]
Stage C+	3.32%	[42]
Stage D	3.28%	Assumption
Mortality	5.43%	[42]
Percent of Heart Failure Hospitalizations that are Preventable	54.20%	[16]
50% reduction in preventable hospitalization for SENC	27.10%	Assumption
HF hospitalization (annualized rate)	32.02%	[17]
30-day HF rehospitalization	26.90%	[43]

**Table 2:** Resource Use Input Data for Payer Model

Variables	Base-case	Article References
Fast-SENC MRI rest	$324	Medicare Fee Schedule
ECHO rest	$231	Medicare Fee Schedule
Contrast medicine	$45	Assumption
Drug costs PMPM for HF patients	$367	[44]
Drug costs for advanced HF patients	$580	[44]
Mean cost of HF hospitalization as primary diagnosis	$17,654	[45]
Mean cost of HF hospitalization as secondary diagnosis	$25,325	[45]
Mean cost of HF hospitalization	$23,077	[45]
Cost of Ischemic heart disease hospitalization	$14,989	[45]
Outpatient/Office visit	$ 165	Medicare Data Milliman Report
Cost discount rate	3%	[40]

.

### Key assumptions

The model assumed that patients entered into two hypothetical cohorts when they were assessed for HF with either Fast-SENC or ECHO. With the ability to detect changes and deterioration of the heart wall, Fast-SENC was assumed to be able to allow clinicians to start patients with medical therapy and lifestyle changes. Michalsen et al. concluded that 54.2% of HF hospitalization could be avoidable if patients received treatment sooner, adhered to prescribed medications and changed their lifestyles [16]. Thus, we assumed that using Fast-SENC a 27.1% reduction in HF hospitalizations (i.e., assuming half of Michalsen et al.’s reported avoidable hospitalizations) could be achieved with early HF diagnosis.

The model assumed that disease-state costs do not vary across cohorts, and that at each state the following costs were incurred: (1) Stage B costs included annual physician visits, medication and diagnostic imaging; (2) Stage C costs included probability of intervention procedural costs; (3) Stage C+ included costs of HF hospitalization and the probability of rehospitalization; and (4) Stage D costs included palliative care and the likelihood of mechanical assist device implantation. Total disease state costs were multiplied by the appropriate transition probabilities (1 – transition probability) for each corresponding state to calculate the expected cost across cohorts. Finally, we assumed that the quality of life values did not differ among the cohorts. Average utility for all patients in Stage B heart failure was 0.80 for patients with mild HF, 0.65 for advanced HF patient and 0.30 for all patients in the last stages of the HF disease [35]. Health utilities were multiplied by the percentage of patients alive in each cohort to derive the average quality-adjusted life years (QALYs) for the cohort in each year of the model.

### Hospital model

The hospital model is a linear one-year accounting model estimating total cost of Fast-SENC and ECHO imaging ownership, imaging facility and technical costs, test costs, and medication costs. The hospital model aims to provide per patient hospital contribution margin for all HF patients diagnosed early with either ECHO or Fast-SENC. Fast-SENC costs were calculated using an initial purchasing price for the software, an ongoing maintenance cost, and a per-test fee. The hospital model calculated hospital profit contribution margins per HF diagnostic test, comparing Fast-SENC and ECHO. The model quantified lost annual marginal revenue for patients admitted for unplanned hospitalizations and also quantified the additional contribution margins from scheduled interventions. These interventions included valve procedures, ablation, revascularization and cardiac rhythm management [25, 37, 38]. In sum, the calculation can be expressed as HospContM = HospDiagR – Cdiag * HospHFIncidence, where HospContM refers to hospital contribution margin, HospDiagR is diagnostic procedure reimbursement, Cdiag is cost of diagnostic test, and HospHFIncidence is the annual number of patients with HF treated within a hypothetical U.S. hospital system. Additional downstream effects of early HF diagnosis were also included in the hospital model, and can be summarized in two expressions: (1) HFhospC = ERHosp – PlannedHosp * (HospR – HospC + NonR30dayHosp); and (2) Planned Interventions Revenue = HospHFIncidence * Diagnostic Accuracy * Planned Procedure Rate. In these expressions, HFhospC refers to HF hospitalization cost, ERHosp is emergency hospitalization, PlannedHosp is planned hospitalization, HospR is hospital reimbursement, HospC is hospitalization cost and NonR30dayHosp is non-reimbursable 30-day hospitalization cost.

Procedural costs were equivalent to average payer reimbursement rates, which we conservatively estimated to be equivalent to U.S. Medicare rates. Table 3 provides further detail on clinical and cost input data for the hospital model.

**Table 3:** Hospital model input data

Parameter	ECHO	Fast-SENC Rest	Source
Prevalence of Asymptomatic Heart Failure	20%	20%	Assumption
Patients in High Risk Population to be Imaged	3000	3000	KOL interviews
Stage B	4.27%	3.11%	[42]
Stage C	4.55%	3.32%	[42]
Stage C+	4.50%	3.28%	Assumption
CMS and private payer (90% to10% ratio) reimbursement & cost for HF hospitalization	$14,631	$14,631	[46]
HF hospitalization cost from NIS 2015 sample	$7463	$7463	[47]
30 day HF rehospitalization	26.90%	26.90%	[43]
HF hospitalization	56.00%	N/A	[17]
Imaging Per Year of Identified Asymptomatic HF Patients	2	2	Assumption
ICD	48%	48%	[26]
CRT	20%	20%	[26]
Valve procedures	4.5%	4.5%	[37]
Ablation procedures from tachycardia-induced cardiomyopathies	0.79%	0.79%	[48]
Revascularizations for infarcted patients (previous MI)	4.7%	4.7%	[38]
Total cost of ownership:			
Acquisition cost (one time)	N/A	$40,000	MSI
Maintenance cost (annual)		$10,000	MSI
Assumed medical equipment lifetime (years)	10	10	Assumption
Cost per Test	N/A	$4.67	Calculation
Imaging Modality Parameters:			
Time Occupying Machine (min)	30	12	Hospital procedural guidelines
Imaging Facility Cost Per Hour (Including Overhead, Wages, Bills, etc.)	$200	$320	Market research outcomes
Test Inventory (Per Test)	$0	$150	MSI
Cost of Contrast	$34	$0	CMS Fee Schedule 2017
Physician Reading Fee	$50	$0	Assumption
Reimbursement Parameters:			
Test Reimbursement	$231	$324	2017 Medicare fee schedule
Contrast Reimbursement	$34	$0	CMS 2017

*Note: MSI = Myocardial Solutions Inc*

## Results

### Payer model

The results of the payer model are shown in Table 4. Discounted total life-years and QALYs in the Fast-SENC cohort were 3.05 years, or1.96 QALYs. In the ECHO arm, the total life-years were 1.71, 0.88 QALYs. Over their respective lifetimes, patients in the Fast-SENC cohort accumulated $24,647 (discounted at 3% per year) in healthcare costs compared to substantially higher costs in the EHCO group ($39,097). Thus, the Fast-SENC cohort had lower costs and higher QALYs when compared to the ECHO cohort, and consequently Fast-SENC generated the largest cost savings difference in the first year, costing 72% less than the ECHO arm. This difference narrowed over the years, as the Fast-SENC arm accrued additional costs resulting from a lower mortality rate, yielding a lifetime savings of 37% for using Fast-SENC instead of ECHO. Fast-SENC’s cost effectiveness is dominant to EHCO at all time horizons.

**Table 4:** Payer Model Results

Cumulative per Person	Fast-SENC	ECHO	Difference	Percent Difference
1-Year Horizon	$878	$3172	($2294)	−72%
3-year horizon	$15,031	$33,230	($18,199)	−55%
5-year horizon	$19,319	$36,526	($17,208)	−47%
10-year horizon	$19,154	$38,708	($19,554)	− 51%
Lifetime	$24,647	$39,097	($14,450)	-37%
Life Years	3.1	1.7	1.3	78%
QALY	2.0	0.9	1.1	124%
Cost per QALY	$12,551	$44,623	($32,071)	−72%
Cost per LY	$8078	$22,845	($14,767)	−65%
ICER (SENC vs ECHO)		Savings	($13,288)	per QALY

### Hospital model

The results of the hospital model are presented in Table 5. The total cost per HF patient visit is $184 for ECHO and $209 for Fast-SENC, which results in hospital contribution margins of $81 and $115, respectively. Based on an assumption of 4200 procedures annually utilizing Fast-SENC, a hospital could achieve $481,600 in total contribution margins. The Fast-SENC approach generates $238,600 more in contribution margins when compared to ECHO annual utilization. In addition, Fast-SENC would allow clinicians to plan interventional procedures in advance instead of receiving HF patients in the emergency room. Fast-SENC is likely a more efficient use of resources for hospitals given its shorter exam time compared to ECHO, which allows for a significantly higher hourly profitability contribution. Fast-SENC generated a contribution margin of $573 per hour of testing, while ECHO generated only $162. As a result of its ability to identify early heart failure patients, Fast-SENC increases the number of scans the hospital will execute in the study patient population, as patients diagnosed with heart failure will require additional Fast-SENC tests to monitor and manage their condition.

**Table 5:** Hospital Model Results

Summary Metrics	ECHO	Fast-SENC	Margin gain
Hospital contribution margin per HF imaging	$81	$115	$34
Hospital HF test contribution margin per hour	$162	$573	$411
Annual hospital contribution margin per HF imaging test	$24,300	$481,600	$238,600

### Sensitivity analysis

**Fig. 2:**
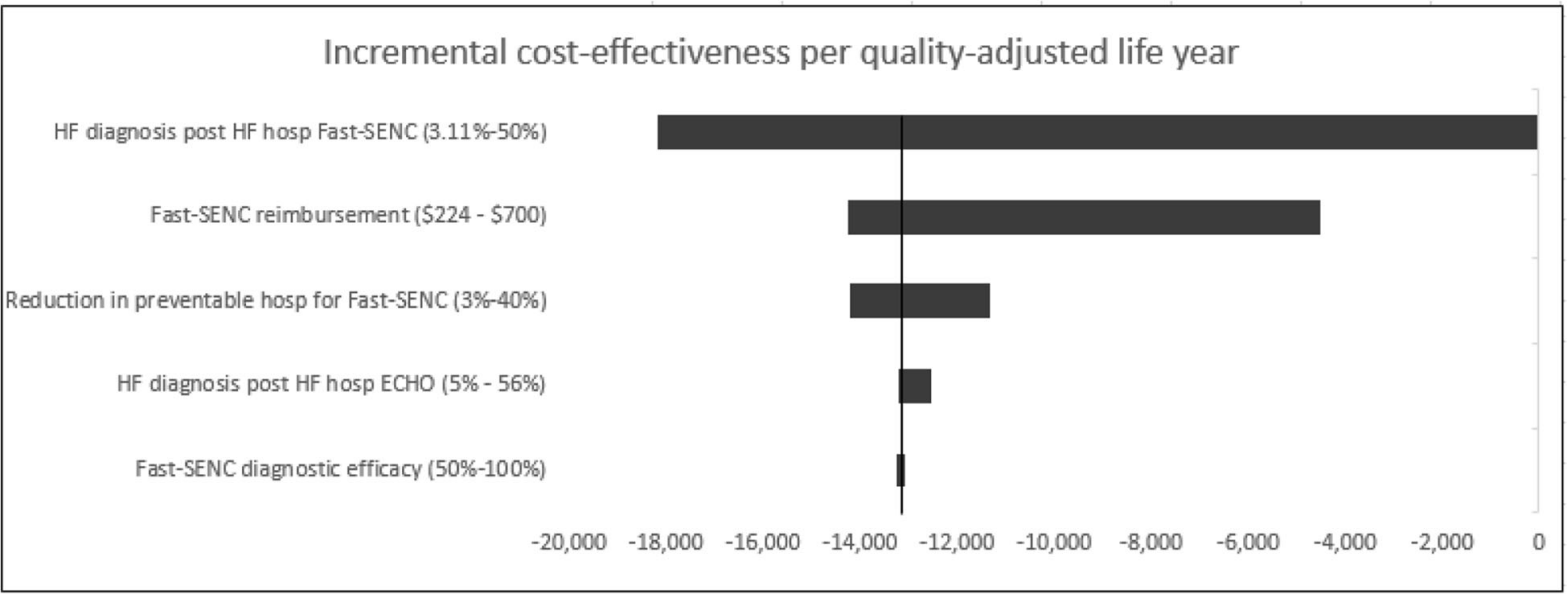
Tornado diagram presenting results of univariate sensitivity analysis. The horizontal axis shows various incremental cost-effectiveness per quality-adjusted life year. At a threshold of $50,000 per QALY, Fast-SENC strategy remains robust for all parameters

We performed sensitivity analysis on key input parameters affecting the cost-effectiveness (payer model) of both patient cohorts. These parameters include, for example, Fast-SENC HF diagnostic accuracy, diagnosis of HF post HF hospitalization event for both patient cohorts, reduction in preventable hospitalization in the Fast-SENC group, Fast-SENC test reimbursement and the rate of HF diagnosis post HF hospitalization in the Fast-SENC group. One-way sensitivity analysis resulted in the tornado diagram shown in Fig. 2. Sensitivity analysis ranges were based on the parameter ranges reported in the literature search [16, 17, 22, 39]. The results of univariate sensitivity analysis showed that the Fast-SENC strategy remained robust for all parameters and did not surpass the threshold of $50,000 per QALY.

## Discussion

In this study, we performed a cost-effectiveness analysis to compare the currently available echocardiography strategy for assessing HF and a newly developed Fast-SENC CMR diagnostic approach. From a payer perspective, we found that Fast-SENC was associated with higher quality-adjusted life years (1.96 v .88) and, over a lifetime, patients in the Fast-SENC cohort accumulated $24,647 per patient in expected healthcare costs compared to the higher costs in the ECHO group ($39,097 per patient). In the first year, Fast-SENC, compared to ECHO, reduced costs by 72%, although the cost-savings difference narrowed to 32% for the lifetime horizon. The driving factor of this apparent decrease in cost savings benefit is the extended lifetime and lower mortality of Fast-SENC arm patients leading to additional costs for care. By increasing the longevity and survival of patients, Fast-SENC’s ability to identify heart failure before patients become symptomatic creates a significant opportunity to monitor and manage patients that otherwise would be identified by a cardiac event. By extrapolating these results to the entire US health system, we estimate the savings potential of Fast-SENC to be over $7 billion annually increasing over time with $19 billion in savings annually by 2030. Actual savings potential could be higher when adjusted for private payer savings.

From the hospital perspective, a hospital can (on average, based on 4200 procedures annually utilizing Fast-SENC) generate $481,600 in contribution margin, which is $238,600 more compared to ECHO. The contribution derives from a combination of a higher profitability per test for Fast-SENC and an increased test volume for monitoring and managing heart failure patients identified by Fast-SENC but missed by ECHO. Fast-SENC’s greater speed and accuracy is believed to impact hospitals and health plans by reducing the frequency of unnecessary procedures and identifying more patients who can benefit the most from more intensive treatment. Fast-SENC’s ability to detect myocardial dysfunction in early heart failure patients can have other beneficial effects for hospitals, including increased ICU bed capacity through reduced HF hospitalizations, decreased 30-day HF patient re-hospitalizations, and better managed HF patients in need of more advanced interventions.

There are a few limitations in our study. The first limitation is the complexity of accurately depicting outcomes and resource use by disease state in simulation models. The second limitation pertains to input data, which were in majority retrieved from prospective studies with inherent uncertainties. However, our sensitivity analysis attempted to identify influential factors and thus describe the scenarios in which our results would be valid. A third limitation is the assumption that disease transition probabilities for the Fast-SENC cohort are reduced because of earlier HF diagnosis and earlier treatment. Finally, based on the Michalsen et al. study that found that 54.2% of all hospitalizations are preventable with better patient management and lifestyle, we assume that Fast-SENC-guided diagnosis could capture half of these hospitalizations (27.1%) through improvement in HF detection and management, though it is possible that this assumption is imprecise. Finally, the assumption that the protective effects of earlier treatment are evident in later disease stages could also be considered a model limitation, as there is little literature based on longer-term longitudinal studies to directly support this assumption. Hence, we based this assumption on communications with clinical experts, who advised that the diagnostic effect could reasonably be tied to the treatment effect. The results are likely to be to some degree sensitive to this assumption.

Further clinical studies are needed to confirm our model assumptions. However, diagnosing HF earlier in the disease progression and treating patients accordingly is a well-studied area with a number of high-quality published studies that conclude increased patient benefits and reduced mortality, similarly to our study. Despite these limitations, this study suggests that diagnostic tests enabling an early discovery of HF with appropriate treatments can be cost-effective if conditions that influence the economic impact of such tests are well evaluated.

## Conclusions

Fast-SENC is associated with higher quality-adjusted life years and lower accumulated expected healthcare costs than echocardiogram patients. Fast-SENC also shows a significant short-term and lifetime cost-savings difference and a higher hospital contribution margin when compared to echocardiography. These results suggest that early discovery of heart failure with methods like Fast-SENC can be cost-effective when followed by the appropriate treatment.

## Additional file

Additional file 1: Transition Matrix Calculations. (DOCX 15 kb)

## Abbreviations

CMR: cardiac-tagged magnetic resonance imaging
ECHO: echocardiography
EF: ejection fraction
HF: heart failure
ISPOR: International Society for Pharmacoeconomics and Outcomes Research
LV: left ventricular
NICE: U.K. National Institute for Clinical Excellence
NYHA: New York Heart Association
QALY: quality adjusted life-years

## Authors’ information

N/A
