# Supplementary material for: Economic evaluation of cardiac magnetic resonance with fast-SENC in the diagnosis and management of early heart failure
Source: Health Econ Rev. 2019 May 23;9:13. doi: 10.1186/s13561-019-0229-7 (PMC6734300; doi:10.1186/s13561-019-0229-7)
Supplement: Supplementary file 1 — Transition Matrix Calculations. (DOCX 15 kb) [file 13561_2019_229_MOESM1_ESM.docx]

| Supplemental Table 1. Transition Matrix Calculation for Myostrain cohort | | | | | | | |
| --- | --- | --- | --- | --- | --- | --- | --- |
|  |  | To | | | | | |
|  |  | Stage B stable | Stage C | Stage C+  (HF Hospitalization) | Stage D | Death | Check |
| From | Stage B stable | 0.718 | 0.031 | 0.033 | 0.025 | 0.193 | 1.000 |
|  | Stage C | 0 | 0.699 | 0.033 | 0.026 | 0.241 | 1.000 |
|  | Stage C+  (HF Hospitalization) | 0 | 0 | 0.490 | 0.033 | 0.477 | 1.000 |
|  | Stage D | 0 | 0 | 0 | 0.250 | 0.750 | 1.000 |
|  | Death | 0 | 0 | 0 | 0 | 1.000 | 1.000 |
| *Note: Matrix calculations are based on the input parameters found in Table 1. | | | | | | | |

| Supplemental Table 2. Transition Matrix Calculation for Echocardiography cohort | | | | | | | |
| --- | --- | --- | --- | --- | --- | --- | --- |
|  |  | To | | | | | |
|  |  | Stage B stable | Stage C | Stage C+  (HF Hospitalization) | Stage D | Death | Check |
| From | Stage B stable | 0.685 | 0.043 | 0.046 | 0.034 | 0.193 | 1.000 |
|  | Stage C | 0 | 0.675 | 0.048 | 0.036 | 0.241 | 1.000 |
|  | Stage C+  (HF Hospitalization) | 0 | 0 | 0.478 | 0.045 | 0.477 | 1.000 |
|  | Stage D | 0 | 0 | 0 | 0.250 | 0.750 | 1.000 |
|  | Death | 0 | 0 | 0 | 0 | 1.000 | 1.000 |
| *Note: Matrix calculations are based on the input parameters found in Table 1. | | | | | | | |
